# Differential Protein Profiling of Cerebrospinal Fluid in Piglets with Severe Meningoencephalitis Caused by *Streptococcus suis* Type 2 Compared to Controls

**DOI:** 10.3389/fcimb.2018.00035

**Published:** 2018-02-09

**Authors:** Hongtao Liu, Li Jia, Wenfei Guo, Yingying Sun, Rining Zhu, Shuguang Li, Guanggang Qu, Hexiang Jiang, Junjie Wang, Jingmin Gu, Changjiang Sun, Xin Feng, Wenyu Han, Liancheng Lei

**Affiliations:** ^1^College of Veterinary Medicine, Jilin University, Changchun, China; ^2^Shandong Binzhou Animal Science and Veterinary Medicine Academy, Binzhou, China

**Keywords:** *Streptococcus suis*, meningoencephalitis, blood-CSF barrier, cerebrospinal fluid, proteomics

## Abstract

*Streptococcus suis* serotype 2 (SS2) is a zoonotic pathogen that can cause meningitis both in pigs and in human beings. However, the pathogenesis of central nervous system (CNS) infection caused by SS2 have not yet been elucidated. To find the key molecules in cerebrospinal fluid (CSF) needed for the pathogenesis, a SS2 meningoencephalitic pig model and a SS2 non-meningoencephalitic pig model were established in this study. CSF was collected from infected piglets, and protein profiling was performed with label-free proteomics technology. A total of 813 differential proteins, including 52 up-regulated proteins and 761 down-regulated proteins, were found in the CSF of meningoencephalitic pigs compared with both non-meningoencephalitic pigs and healthy pigs. These 813 differential proteins were clustered into three main categories, namely, cellular component, biological process, and molecular function by gene ontology (GO) analysis. The most enriched subclasses of differential proteins in each category were exosome (44.3%), energy pathway (25.0%) and catalytic activity (11.3%), respectively. The most enriched subclasses of upregulated proteins were extracellular (62.1%), protein metabolism (34.5%) and cysteine-type peptidase activity (6.9%), and of downregulated proteins were exosomes (45.0%), energy pathway (24.0%) and catalytic activity (9.4%). Then, the differential proteins were further investigated by using the KEGG database and were found to participate in 16 KEGGs. The most enriched KEGG was citrate cycle (56.6%), and some of these differential proteins are associated with brain diseases such as Huntington's disease (18.6%), Parkinson's disease (23.8%) and Alzheimer's disease (17.6%). Sixteen of the 813 differential proteins, chosen randomly as examples, were further confirmed by enzyme-linked immunosorbent assay (ELISA) to support the proteomic data. To our knowledge, this is the first study to analyze the differential protein profiling of CSF between SS2 meningoencephalitic piglets and non-meningoencephalitic piglets by employing proteomic technology. The discovery and bioinformatics analysis of these differential proteins provides reference data not only for research on pathogenesis of SS2 CNS infection but also for diagnosis and drug therapy research.

## Introduction

*Streptococcus suis* type 2 is a zoonotic pathogen that can cause meningitis, bacteremia, septicemia, endocarditis, and arthritis in pigs and humans. Meningitis is the most serious symptom of *S. suis 2* infection and often shows clinical symptoms including vertigo, ataxia, headaches, neck stiffness, fevers, nausea, deafness and so on (Madsen et al., 2002; Mai et al., 2008; van Samkar et al., 2015; Dejace et al., 2017; Sena et al., 2017), and histopathological features including the presence of fibrin, edema and cell infiltration in the meninges and choroid plexus. As a result, this pathogen poses a great threat not only to the swine industry but also to human health.

Obtaining effective and controllable drugs that can pass through the blood-CNS barrier to treat neurological diseases remains a challenge for modern medicine (Coureuil et al., 2017). Understanding the pathogenesis of SS2 meningitis is critical for identifying candidate targets for the diagnosis and therapy of SS2 meningitis. SS2 can cause meningitis due to the interaction between SS2 virulence factors and host molecules. Therefore, not only do the virulence factors from SS2 play an important role in the process of SS2 causing meningitis, but host molecules are also very important for this process.

The blood-CNS barrier, including BCB (blood-CSF barrier) and BBB (blood-brain barrier), is important for maintaining CNS homeostasis. The prerequisite of SS2 causing meningitis is SS2 penetration into the blood-CNS barrier. The blood–CSF barrier is composed of three interfaces: the choroid plexuses (CPs), the pial microvessels and the arachnoid barrier cell layer (Brochner et al., 2015). The CSF is secreted by the CPs, which are villous structures attached to the ventricular ependyma (Strazielle and Ghersi-Egea, 2000). Many studies have reported that some molecules in CSF could be used as potential biomarkers of some brain diseases and bio-targets of therapeutic drugs and pathogenesis research. For example, the porcine cathelicidin PR-39 was found to be significantly increased in CSF of piglets with meningitis and could inhibit neutrophil DNA degradation by bacterial nucleases (de Buhr et al., 2017). Insulin concentrations often found to be decreased in CSF of Alzheimer's disease (AD) patients and could providing insights for AD pathogenesis research (Craft et al., 1998). Comprehensive proteome profiles of different grades of gliomas using CSF could providing insights into disease pathobiology and differentially abundant CSF proteins may serve as potential disease monitoring and prognostic markers for gliomas (Gahoi et al., 2017). The findings of these studies suggested that the discovery of the differential protein in CSF could provide important candidate biological targets for the research of diagnosis, pathogenesis and drug therapy of brain diseases.

Label-free quantification is a method in mass spectrometry (MS). Unlike other methods for protein quantification, label-free quantification does not use a stable isotope to chemically bind to and thus label the protein. This method was widely used to determine the relative amount of proteins in two or more biological samples (Bantscheff et al., 2007; Asara et al., 2008). UniProt is a freely accessible database of protein sequence and functional information, which was built by UniProt consortium that comprises the European Bioinformatics Institute (EBI), the Swiss Institute of Bioinformatics (SIB), and the Protein Information Resource (PIR), many entries being derived from genome sequencing projects. It contains a large amount of information about the biological function of proteins derived from the research literature (O'Donovan et al., 2002; Boeckmann et al., 2003; Wu et al., 2003).

There are no previous reports about the protein profiling of cerebrospinal fluid in meningitic pigs. For this purpose, SS2 meningoencephalitic and non-meningoencephalitic pig models were established in this study. CSF from different pig models was collected and analyzed by protein profiling using label-free proteomics technology. The data of proteomic analyses are useful for research of SS2 CNS infection diagnosis, pathogenesis and therapy.

## Materials and methods

### Ethics statement

Bama miniature pigs used for infection experiments were purchased from Shandong Binzhou Animal Science Veterinary Medicine Academy. All animal experimental procedures were performed in strict accordance with the Regulations for the Administration of Affairs Concerning Experimental Animals approved by the State Council of the People's Republic of China (1988.11.1).

### Bacterial strains and culture conditions

SS2 strains used in this study were isolated from sick pigs. The JZLQ022 strain was isolated from brain tissue of meningitis pig, and the JZLQ001 strain was from lymphonodi mandibulares of arthritis pig. SS2 strains were grown on tryptic soy agar (TSA) plates with 5% newborn bovine serum (CLARK Co. Ltd., Australia) for 10 h at 37°C. Then isolated colonies were transferred into 3 ml of tryptic soy broth (TSB) with 5% newborn bovine serum and incubated for 8 h at 37°C with agitation. The bacterial pellet was obtained by centrifugation and resuspended in phosphate-buffered saline (PBS) with a continuous 10-fold dilution. These dilutions were plated onto TSA plates to accurately determine the CFU per milliliter.

### The establishment of a porcine model of SS2 infection

Eighteen 26-day-old healthy inbred Bama miniature pigs were randomly divided into three groups (*n* = 6): meningoencephalitis group (200 μL PBS with 5 × 10^6^ CFUs JZLQ022), non-meningoencephalitis group (200 μL PBS with 5 × 10^6^ CFUs JZLQ001) and healthy group (200 μL PBS) by ear intravenous injection. Clinical symptoms including appetite, mental status and body temperature were continuously monitored post infection.

### Collection of samples and determination of viable bacteria in organs

Heparinized blood was collected 2 h, 4 h, 12 h, 2 d, 3 d, 5 d, and 7 d after infection. The pigs were euthanized with sodium pentobarbital (400 mg/mL at a dose of 500 μL/kg) by ear intravenous injection when pigs were dying. After euthanasia, skin and muscles in the back of neck was cut and foramen magnum was exposed. 10 ml injector was inserted into the foramen magnum keep away from spinal cord to collect the CSF. Brain tissue was collected after opening the cranial cavity. 1 g of brain tissue was made into homogenate with 500 μL PBS and plated onto TSA plates to accurately determine the CFU per gram.

### Liquid chromatography-mass spectrometry (LC-MS/MS) measurements

The CSF samples were added to appropriate doses of lysis buffer (7 M Urea, 2 M Thiourea, 0.1% CHAPS) and vortex blended followed by incubation for 30 min s at 25°C. Then, the sample was centrifuged at 15,000 g for 20 min at 4°C. The supernatant was subpackaged into a 1.5 mL tube. The concentration of total protein in the supernatant was determined by Bradford method (Bradford, 1976). LC-MS/MS was performed at the QLbio Biotechnology Corporation in Beijing using the Thermo Scientific EASY-nLC 1000 System and Thermo Q-Exactive MS system. Raw data were identified by Maxquant software and the Sus scrofa protein database on the Uniprot Website (http://www.uniprot.org/).

### Proteomic data analysis

Fold changes in proteins were determined according to label-free quantitative (LFQ) intensity between the meningitic group and non-meningitic group or healthy group. Proteins with a difference >1.5-fold or < 0.66-fold, *p*-values < 0.05 were regarded as differentially abundant proteins. Functional analysis of differential proteins was performed based on the gene ontology (GO) annotations (http://www.geneontology.org/), and the proteins were categorized according to their cellular components, molecular functions and biological processes (http://pantherdb.org/geneListAnalysis.do). The differential proteins were further assigned to the Kyoto Encyclopedia of genes and genomes (KEGG) database (http://kobas.cbi.pku.edu.cn/anno_iden.php).

### Validation with enzyme-linked immunosorbent assay (ELISA) analyses

Some differential proteins in CSF from differential infected piglets, including 7 up-regulated proteins (CTSL1, CTSZ, SAA, MMP-2, RBP-4, CHGA, and CuZnSOD) and 9 down-regulated proteins (Rock, Clathrin, Vinculin, Arp2/3, RhoA, Bid, CytC, PKC, and ERK), were detected by double-antibody sandwich ELISA kits (Purchased from Shanghai Jin Ma Biological Technology co., LTD).

## Results

### Establishment of a pig model of SS2 meningoencephalitis

**Figure 1:**
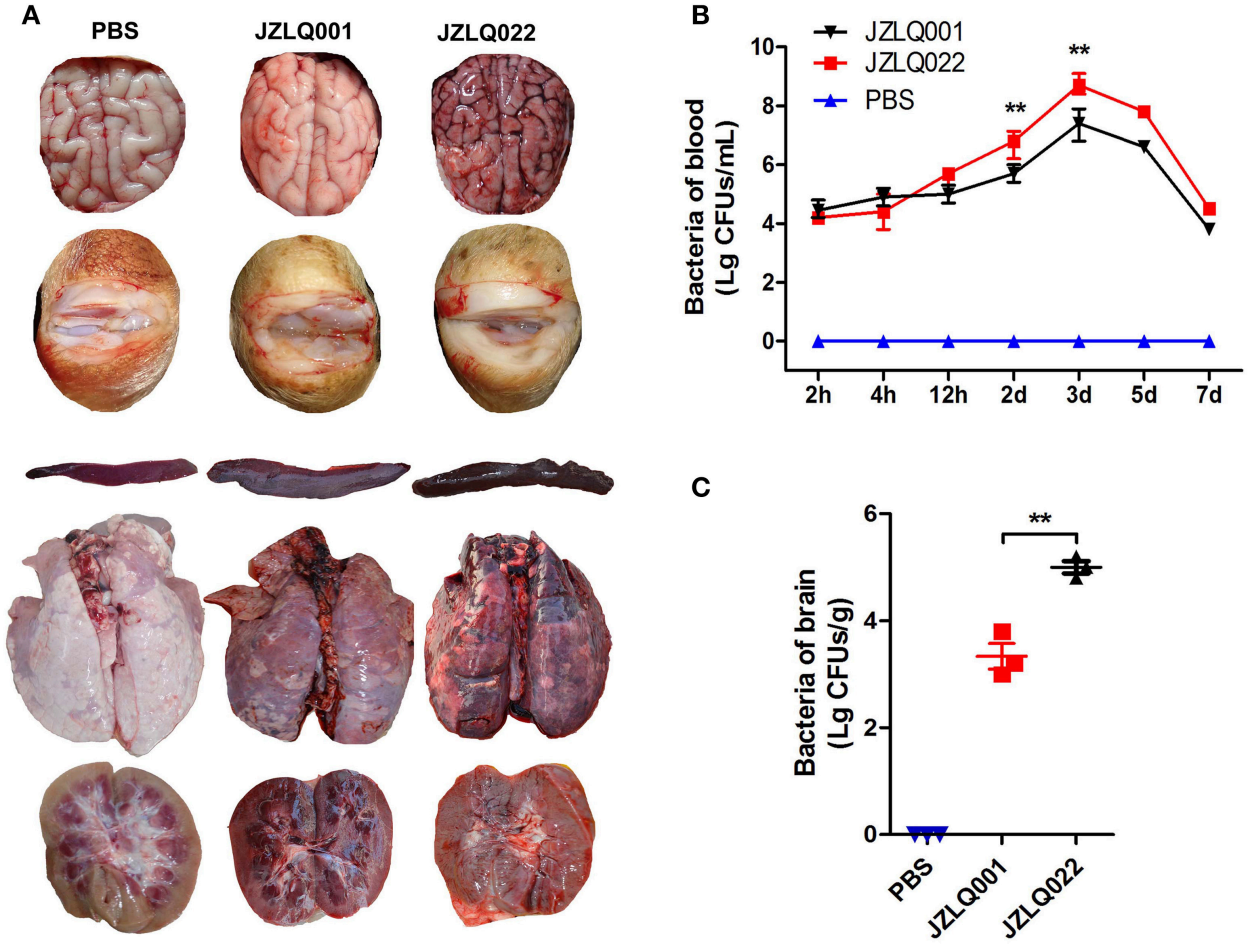
Establishment of SS2 meningoencephalitis and non-meningoencephalitis pig models. **(A)** Autopsy results of brain and arthrosis; (Control: pig infected with PBS representing the healthy group; JZLQ001: pig infected with the JZLQ001 strain representing the non-meningoencephalitic group; JZLQ022: pig infected with the JZLQ022 strain representing the meningoencephalitic group). **(B)** Bacteria counts in blood collected at different time points after infection with SS2 (*n* = 6; ^**^p < 0.01). **(C)** Bacteria counts in brain tissue collected at different time points after infection with SS2 (*n* = 3; ^**^p < 0.01).

Decreased appetite and depressed emotion were observed both in the two groups of pigs infected with JZLQ022 and JZLQ001, respectively. Hyperpyrexia was monitored, and the body temperature of some pigs in the two groups was up to 40°C at 24 h. Arthritis symptoms, including limp, trouble walking and arthrocele, were observed in some pigs infected with JZLQ001 at 3 d (Supplementary Material Video 1). Trouble walking was also observed in some pigs infected with JZLQ022 at 4 d, and foaming at the mouth and ataxia was observed at 5 d (Supplementary Material Videos 2, 3). By autopsy, conspicuous macroscopic lesions, including lung purulent lesions, spleen swelling and infarction, and kidney nephremia, were observed both in the JZLQ022 and JZLQ001 groups (Figure 1A). In addition, joint effusion was observed in the JZLQ001 group. In accordance with the macroscopic lesions, brain hyperemia and hemorrhage were observed in the JZLQ022 group, but not in the JZLQ001 group (Figure 1A). Collected blood and brain tissues were used for bacterial counting. The JZLQ022 infection group had higher SS2 CFUs than PBS control group and the JZLQ001 group in blood at 2 d, 3 d (Figure 1B) and in brain tissues (Figure 1C). The pig models of meningoencephalitis and non-meningoencephalitis were successfully established.

### Comprehensive analysis of proteomic data

**Figure 2:**
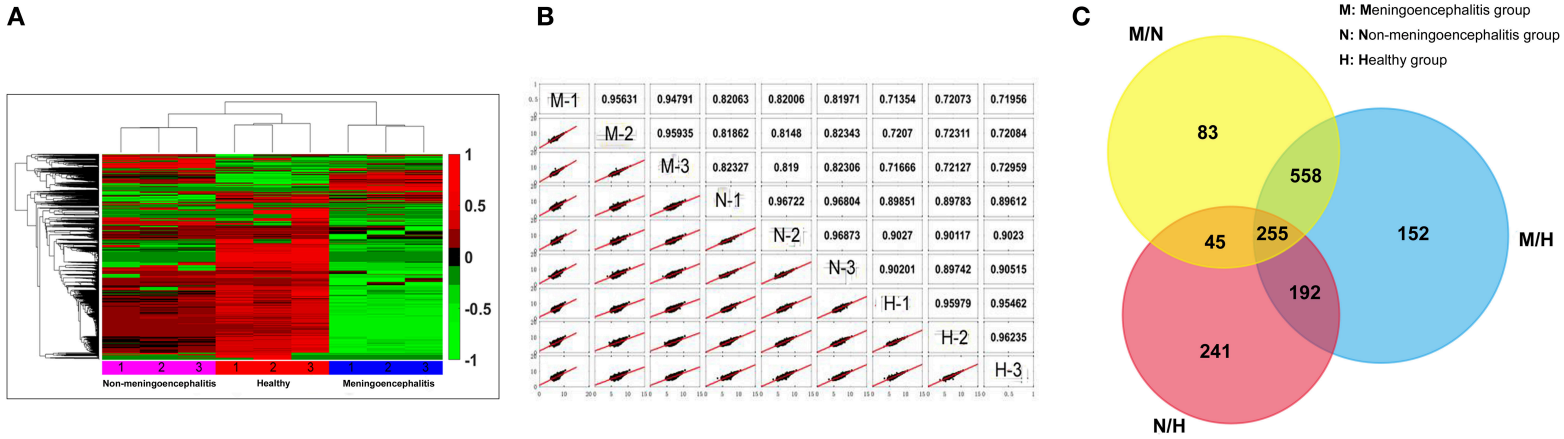
Comprehensive analytic results of proteomic data. **(A)** The heat-map of proteomics results. (Red, 1: up-regulated proteins; Green, −1: down-regulated proteins; Black, 0: Unchanged proteins). **(B)** Repetitive analysis of proteomic data. (M, meningoencephalitic group; N, non-meningoencephalitic group; H, Healthy group). **(C)** Venn diagram of the number of differential proteins among the three treatment groups. M/N, Differential proteins between the meningoencephalitic group and non-meningoencephalitic group; M/H, Differential proteins between the meningoencephalitic group and healthy group; N/H, Differential proteins between the non-meningoencephalitic group and healthy group.

The CSF of pigs from the three treatment groups was collected and analyzed by label-free proteomics technology at the QL-bio Biotechnology Limited Company (Beijing, China). Each sample was analyzed in triplicate. A total of 3456 proteins were authenticated with LC-MS/MS technology and calculated by iBAQ and LFQ algorithms with Maxquant software. The notes of these proteins can be found in Uniprot database. The analysis results show good reproducibility based on the heat-map (Figure 2A) and similarity (the more approximate the value is to 1, the higher the similarity between the two sets of data will be) (Figure 2B). Most molecules in CSF from the meningitic group present lower detection concentrations (green) compared with the other two groups, and a small number of molecules present high detection concentrations (red) (Figure 2A).

Comparison of the changes in proteins between pairs of groups resulted in some similarities and considerable differences. The Venn diagram shows the distribution of the proteins between each pair of groups. Differentially expressed proteins were detected between each pair of groups. Among these proteins, 255 proteins were differentially distributed for all three comparisons (meningoencephalitis vs. non-meningoencephalitis, M/N; meningoencephalitis vs. healthy, M/H; non-meningoencephalitis vs. healthy, N/H). A total of 447 (255 plus 192) co-differential proteins were found when analyzing M/H against N/H. These 447 proteins may provide hints for SS2 infection related research. A total of 813 (255 plus 558) co-differential proteins were found when analyzing M/N against M/H (Figure 2C). These 813 proteins may provide hints for SS2 meningitis related research and are called 813CDPs in the following.

### GO analysis and classification of the 813CDPs

**Figure 3:**
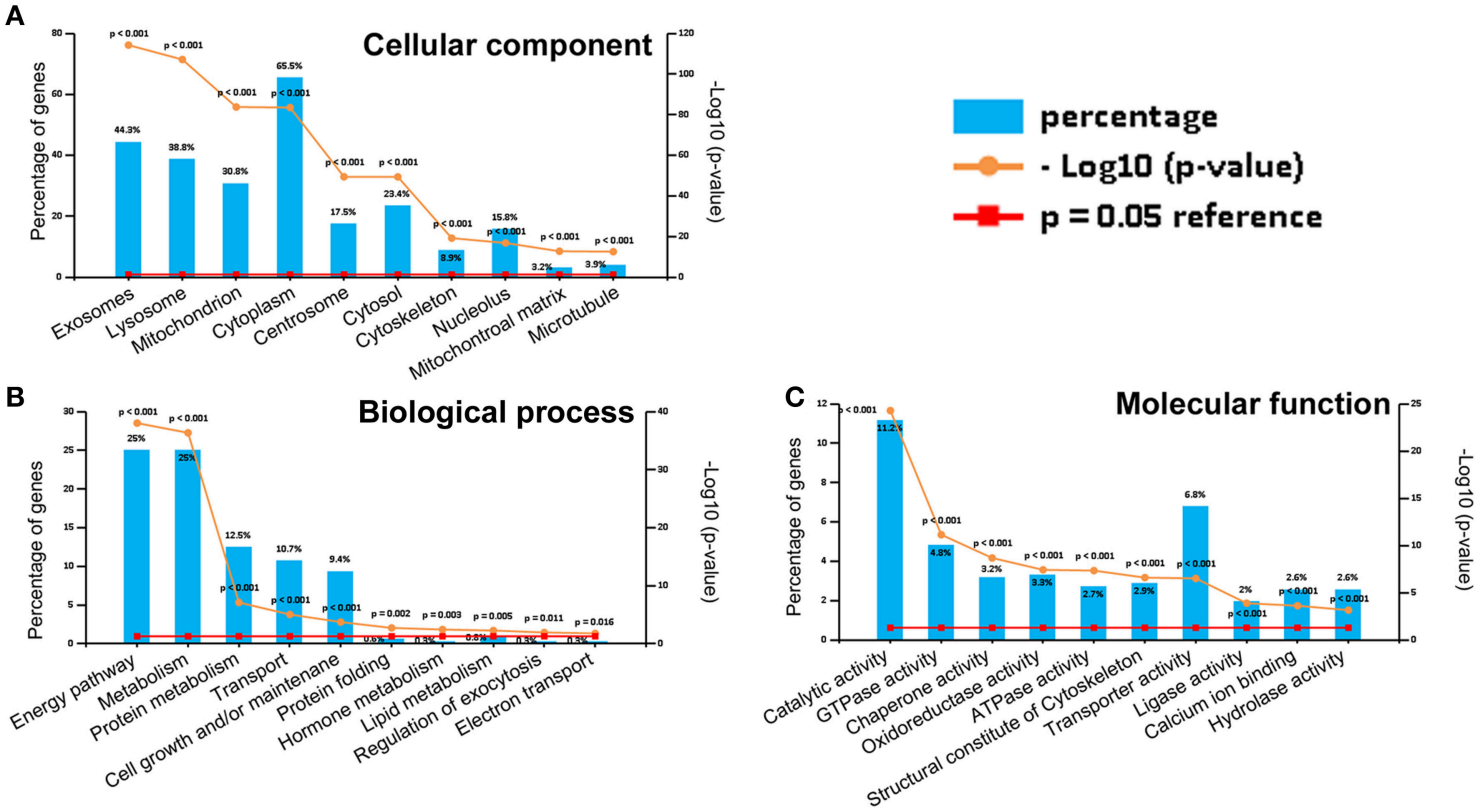
Gene ontology (GO) analysis of 813CDPs. **(A)** Percentage and *p*-value of proteins in cellular component. **(B)** Percentage and *p*-value of proteins in biological process. **(C)** Percentage and *p*-value of proteins in molecular function.

The 813CDPs were clustered into three main categories, namely, biological process, cellular component, and molecular function, by gene ontology (GO) analysis. The top 10 most enriched GO terms were selected in each category. The 10 GO terms in cellular component contained exosomes (44.3%), lysosome (38.8%), mitochondrion (30.8%), cytoplasm (65.5%), centrosome (17.5%), cytosol (23.4%), cytoskeleton (8.9%), nucleolus (15.8%), mitochondrial matrix (3.2%), and microtubule (3.9%) (Figure 3A). The 10 GO terms in cellular biological process contained energy pathway (25%), metabolism (25%), protein metabolism (12.5%), transport (10.7%), cell growth and/or maintenance (9.4%), protein folding (0.6%), hormone metabolism (0.3%), lipid metabolism (0.8%), regulation of exocytosis (0.3%) and electron transport (0.3%) (Figure 3B). The 10 GO terms in molecular function contained catalytic activity (11.2%), GTPase activity (4.8), chaperone activity (3.2%), ATPase activity (2.7%), structural constitute of cytoskeleton (2.9%), transporter activity (6.8%), ligase activity (2%), calcium ion binding (2.6%), and hydrolase activity (2.6%) (Figure 3C). The 813CDPs contain 52 up-regulated proteins (Table 1, Supplementary Material Data Sheet 1) and 761 down-regulated proteins (Supplementary Material Data Sheet 2). The most enriched subclasses of upregulated proteins were extracellular (62.1%), protein metabolism (34.5%) and cysteine-type peptidase activity (6.9%), and of downregulated proteins were exosomes (45.0%), energy pathway (24.0%), and catalytic activity (9.4%) (Supplementary Material Data Sheet 1). The 52 up-regulated proteins contain 27 characterized proteins and 25 uncharacterized proteins (Table 1).

**Table 1:** Proteins with special higher LFQ intensity value in meningoencephalitis group.

**Uniprot ID**	**iBAQ intensity**	**Fold (M/N)**	**Fold (M/H)**	**Protein name**	**Gene name**
P02189	1173900000	14.72	2.22	Myoglobin	MB
P20112	89256000	2.19	2.73	SPARC	SPARC
F1SC80	210150000	3.12	17.30	Retinol-binding protein 4	RBP4
P79263	6054900000	3.87	5.09	Inter-alpha-trypsin inhibitor heavy chain H4	ITIH4
Q28944	130630000	2.50	7.64	Cathepsin L1	CTSL1
Q29073	12800000	2.00	2.42	Prostaglandin reductase 1	PTGR1
Q5S1S4	120470000	4.58	5.54	Carbonic anhydrase 3	CA3
Q7M329	75512000	2.19	11.90	Ribonuclease T2	RNASET2
I3LRJ4	56294000	2.11	3.30	Vitamin K-dependent protein C	PROC
F1SD66	40023000	3.09	5.46	Chromogranin-A	CHGA
F1SH92	1744300000	4.84	5.78	Inter-alpha-trypsin inhibitor heavy chain H4	ITIH4
A0A0A7BZH3	59116000	4.46	4.66	MHC class I antigen	SLA-1
F2Z5M9	3079600	2.45	0.85	Signal recognition particle 54 kDa protein	SRP54
A0A0B8RSP0	37629000	2.64	3.33	Legumain	LGMN
A0A0B8S096	67002000	2.70	6.056	Dystroglycan 1	DAG1
A1XQT6	155440000	35.36	3.29	MLC1f	MYL1
A5GFX7	20490000	9.61	9.78	Cathepsin Z	CTSZ
A6N9J9	172710000	3.73	6.59	Secreted phosphoprotein 1	SPP1
A7YX24	776620000	2.65	3.70	Gamma-synuclein	SNCG
A8D737	65674000	2.40	5.26	T-cadherin	*N/A*
B5KJG2	55810000	8.35	3.37	Phosphoglycerate mutase	PGAM2
F1RI57	203610000	2.82	4.82	Beta-1,3-N-acetylglucosaminyltransferase	LFNG
K7GQL2	11579000	4.20	90.59	Coagulation factor XIII, A1 polypeptide	LOC100153504
Q007T6	57101000	3.60	11.76	Superoxide dismutase [Cu-Zn]	*N/A*
Q28936	329650000	3.81	6.62	Fibrinogen A-alpha-chain	*N/A*
Q2HXZ9	112470000	10.30	18.27	Serum amyloid A protein	LOC733603
T2HRE7	47316000	4.71	3.83	MHC class I antigen	SLA-2
A0A075B7I9	854990000	36.59	2.66	Uncharacterized protein	*N/A*
F1RMC1	52999000	4.81	3.69	Uncharacterized protein	DMTN
F1RF11	19736000	2.94	3.82	Uncharacterized protein	MMP2
F1RN59	23221000	6.01	23.63	Uncharacterized protein	PAM
F1RNB8	107450000	2.67	3.37	Uncharacterized protein	*N/A*
F1RPQ3	16095000	2.44	3.16	Uncharacterized protein	PSMD14
F1RQP6	11818000	3.23	2.57	Uncharacterized protein	NRXN2
F1RTP0	34001000	2.87	42.97	Uncharacterized protein	N/A
F1RTR7	58848000	2.84	3.41	Uncharacterized protein	YOD1
F1RUQ0	726580000	2.22	10.99	Uncharacterized protein	JCHAIN
F1RVH7	111420000	2.13	3.08	Uncharacterized protein	IGFBP7
F1RW32	22821000	2.67	5.53	Uncharacterized protein	SPARCL1
F1S3H9	8261500	27.27	8.86	Uncharacterized protein	LOC100517145
F1S604	29484000	6.49	3.12	Uncharacterized protein	*N/A*
F1S8V7	21334000	2.77	6.84	Uncharacterized protein	CPN1
F1SCC6	5888200000	2.67	3.04	Uncharacterized protein	LOC100153899
F1SCC7	8583000000	2.02	2.65	Uncharacterized protein	LOC100156325
F1SI48	74075000	2.85	6.00	Uncharacterized protein	EPB42
F1SJY2	85340000	91.08	38.89	Uncharacterized protein	TMX2
F1SQX9	856190000	4.918	4.44	Uncharacterized protein	APOD
F1SUU7	54204000	2.06	3.97	Uncharacterized protein	DDI2
I3LBZ1	268100000	2.29	3.51	Uncharacterized protein	*N/A*
I3LEB3	788240000	2.25	4.75	Uncharacterized protein	PCSK1N
I3LGD9	11528000	6.26	6.21	Uncharacterized protein	AGRN
I3LNM9	83333000	2.87	38.28	Uncharacterized protein	LOC100624077

*Fold (M/N) means LFQ intensity fold change of protein abundance between the meningoencephalitic group and non-meningoencephalitic group; Fold (M/H) means LFQ intensity fold change of protein abundance between the meningoencephalitic group and healthy group*.

### Analysis of highly enriched kyoto encyclopedia of genes and genomes (KEGG)

**Figure 4:**
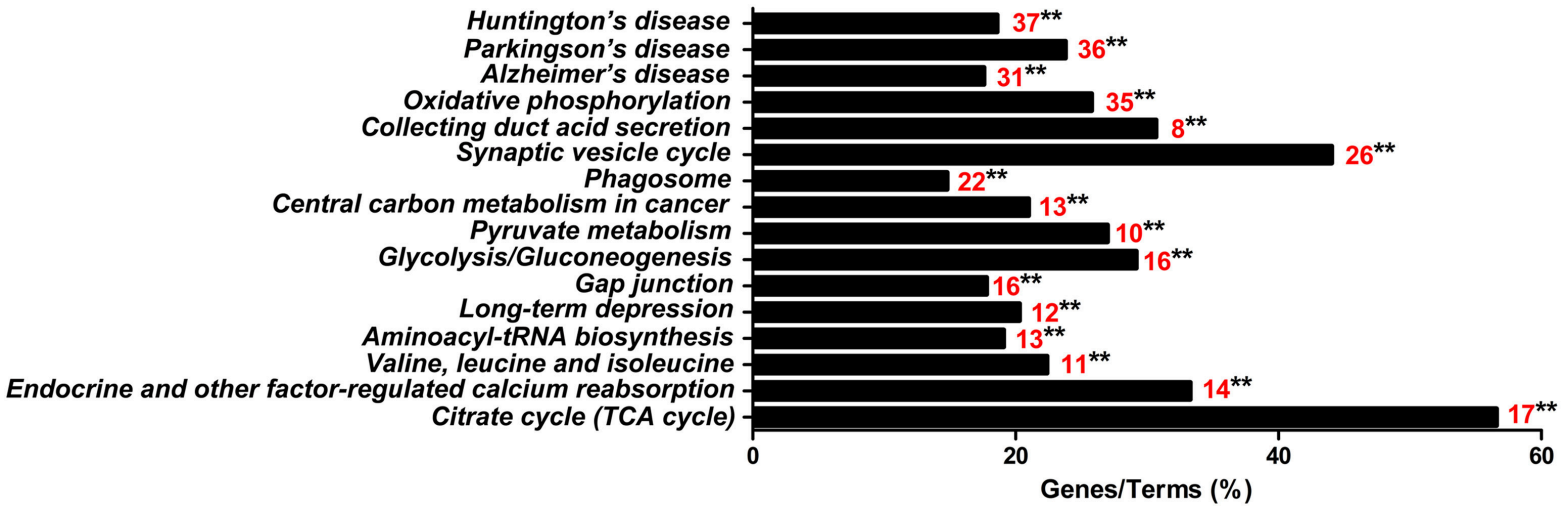
KEGG analysis of the 813 differential proteins. Red number indicates the number of proteins associated with each pathway (^**^p < 0.01).

**Figure 5:**
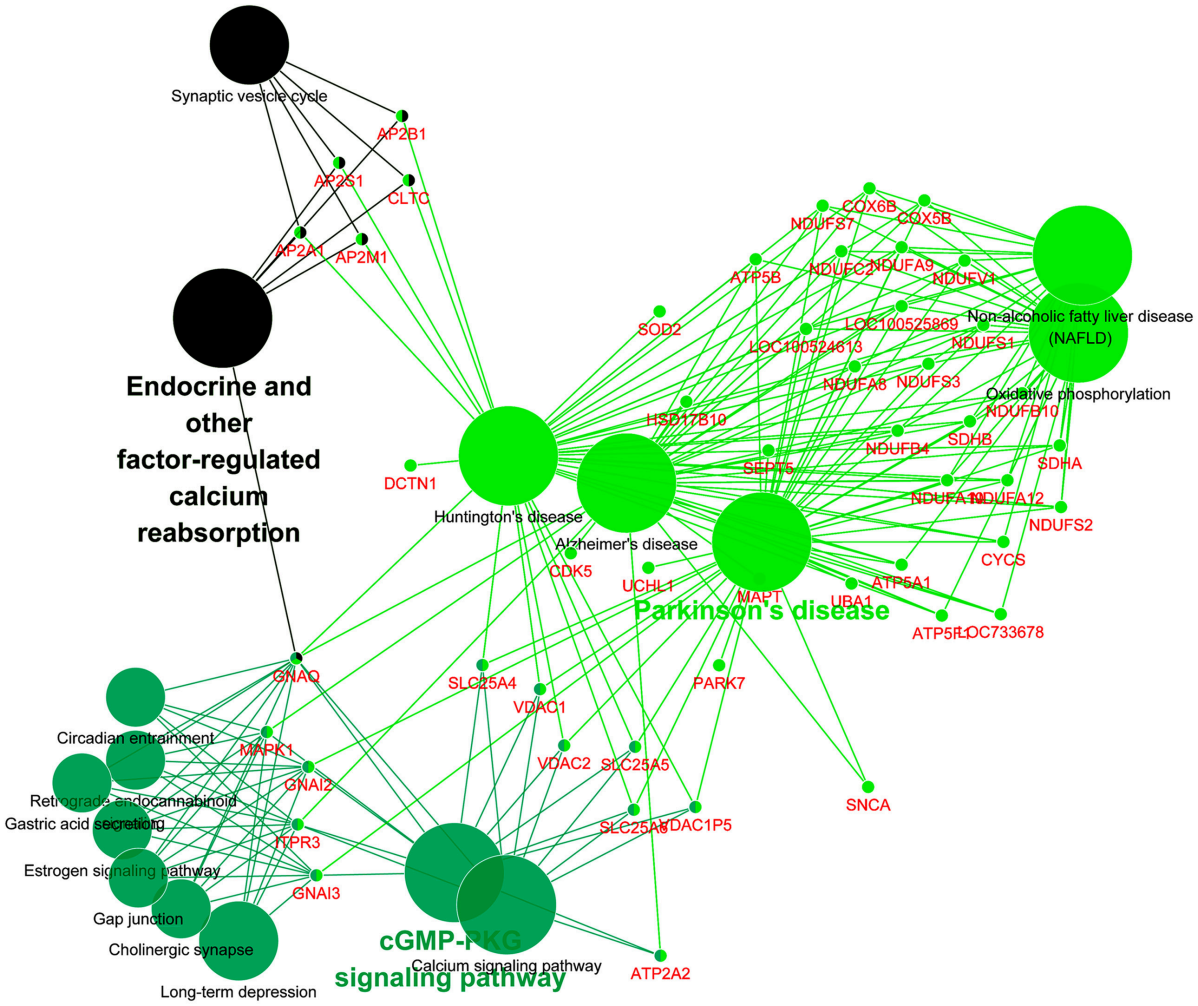
KEGG analysis of differential proteins involved in neurological diseases. Each small dot represents a gene, and each large dot represents a pathway.

The 813CDPs were further investigated by using the KEGG database and were found to participate in 16 KEGGs (Figure 4, Supplementary Material Data Sheet 3). The most enriched KEGG was citrate cycle (56.6%). Interestingly, 49 of 813CDPs are associated with neurological diseases such as Huntington's disease (18.6%), Parkinson's disease (23.8%) and Alzheimer's disease (17.6%). And 23 of 49 differential proteins (genes name: ATP5A1, ATP5B, ATP5F1, COX5B, COX6B, CYCS, LOC100524613, LOC100525869, LOC733678, NDUFA10, NDUFA12, NDUFA8, NDUFA9, NDUFB10, NDUFB4, NDUFC2, NDUFS1, NDUFS2, NDUFS3, NDUFS7, NDUFV1, SDHA, SDHB) are co-exsit in KEGGs of the three neurological diseases (Figure 5). The other enriched KEGGs were Oxidative phosphorylation (25.8%), Collecting duct acid secretion (30.7%), Synaptic vesicle cycle (44.05%), Phagosome (14.8%), Central carbon metabolism in cancer (21%), Pyruvate metabolism (27%), Glycosis/Gluconeogenesis (29.2%), Gap junction (17.8%), Long-term depression (20.3%), Aminoacyl-tRNA biosynthesis (19.1%), Valine, leucine and isoleucine degradation (22.4%) and Endocrine and other-factor regulated calcium reabsorption (33.3%) (Figure 4).

### Verification of a subset of differential proteins by ELISA

**Figure 6:**
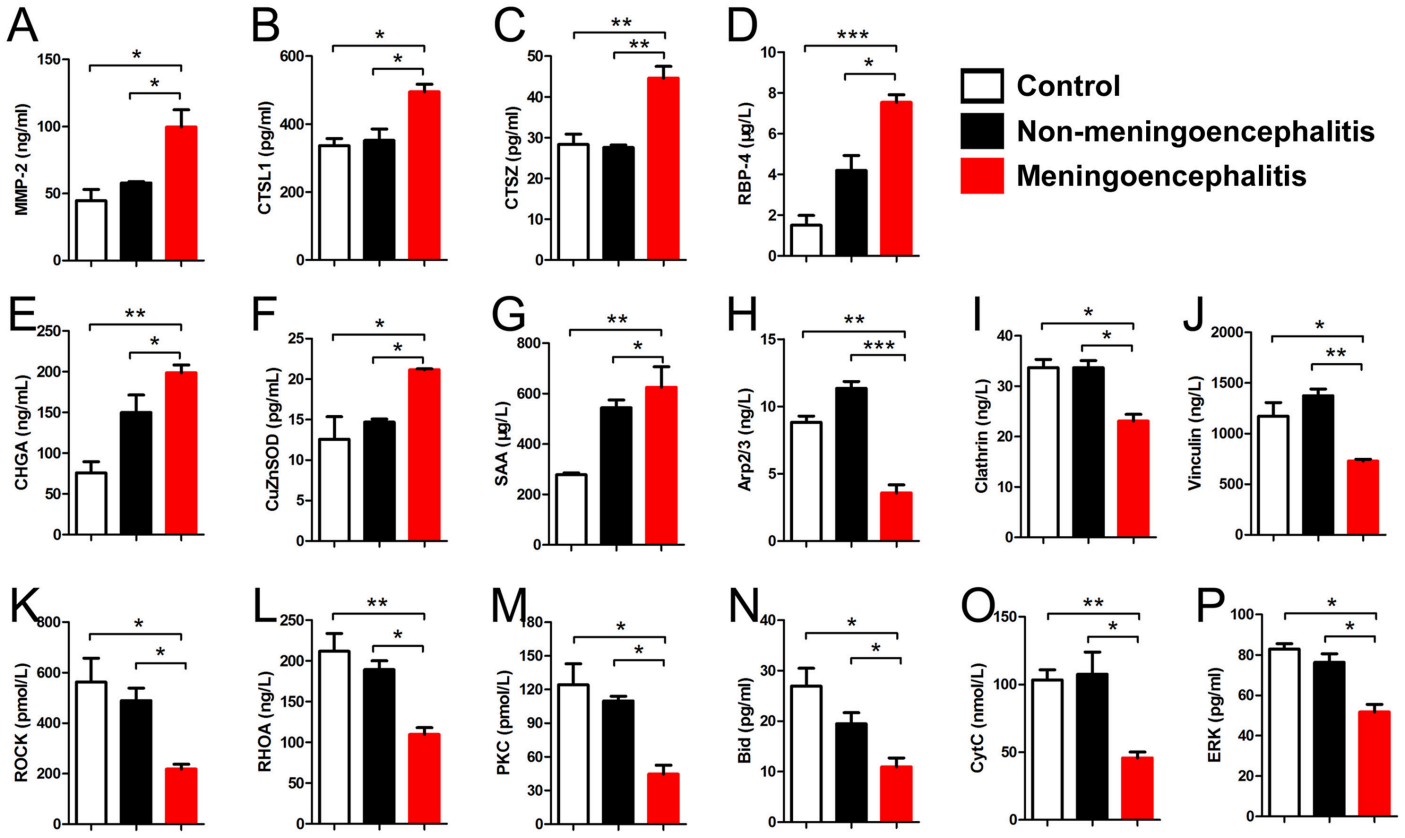
Detection results of differential proteins in the meningitis group by ELISA. **(A–G)** Special up-regulated proteins in meningoencephalitis group. **(H–P)** Special down-regulated proteins in meningoencephalitis group. ^*^p < 0.05; ^**^p < 0.01; ^***^p < 0.001.

According to the previous reports, some differential proteins were selected to be identified by ELISA (*n* = 6). The detection results for 7 up-regulated proteins (CTSL1, CTSZ, SAA, MMP-2, RBP-4, CHGA, CuZnSOD) and 9 down-regulated proteins (Rock, Clathrin, Vinculin, Arp2/3, RhoA, Bid, CytC, PKC, ERK) are in accord with the results of proteomic analysis (Figure 6).

## Discussion

The SS2 meningoencephalitic pig model was successfully established in this study, and differential protein profiling of CSF in meningoencephalitic, non-meningoencephalitic and healthy pigs was analyzed by label-free proteomic analysis technology. A total of 813 co-differential proteins were found when analyzing the meningoencephalitic group with the non-meningitic group against the meningoencephalitic group with the healthy group. Seven up-regulated proteins and 9 down-regulated proteins of these 813 co-differential proteins were further confirmed by ELISA to support the proteomic data. The data provided important hints for research associated with SS2 meningoencephalitis.

The JZLQ022 strain was isolated from brain tissue of meningoencephalitis pig, and the JZLQ001 strain was from lymphonodi mandibulares of arthritis pig. In our pre-experiments, we found that JZLQ022 mainly cause meningitis and JZLQ001 mainly causes arthritis in pigs. Therefore, two SS2 strains, JZLQ022 and JZLQ001, were used to build the meningoencephalitic pig model and non-meningoencephalitic pig model respectively. The model used in this study was a hemorrhagic meningoencephalitis based on results of autopsy (Figure 1A), JZLQ022 strain not only induced meningitis, but also induced severe meningoencephalitis and hemorrhagic encephalitis by crossing BBB and/or BCB. So the differences between the two strains should be analyzed in the future which would provide important implications on mechanism of SS2 across BBB and/or BCB.

Seven up-regulated proteins (CTSL1, CTSZ, SAA, MMP-2, RBP-4, CHGA, and CuZnSOD) in the CSF of meningoencephalitic piglets compared with both non-meningoencephalitic and healthy piglets were found and confirmed by ELISA. One of these proteins, matrix metalloproteinase-2 (MMP-2), is also called 72 kDa type IV collagenase or gelatinase A (Devarajan et al., 1992). There are no previous reports about the relationship between levels of CSF MMP-2 and bacterial meningitis. However, previous reports show that increased levels of serum MMP-2 may reflect the degree of damage to the BCB in bacterial meningitis (Kanoh et al., 2008). Analysis of MMP levels in the CSF indicates early tumor recurrence and can be used to diagnose CNS tumors, such as malignant astrocytomas, brain metastases, and carcinomatous meningitis (Friedberg et al., 1998). Cathepsin Z (CTSZ) is a member of the cysteine cathepsin protease family (Turk et al., 2012) and cathepsin L1 (CTSL1) is a lysosomal cysteine protease that plays a major role in intracellular protein catabolism. There are no reports on the role of CTSZ and CTSL1 in SS meningitis. However, cathepsin B in CSF from patients has been reported to be associated with neurologic diseases (Nagai et al., 2000). Superoxide dismutase [Cu-Zn], also known as superoxide dismutase 1 or SOD1, is one of three superoxide dismutases in humans. It is implicated in apoptosis and amyotrophic lateral sclerosis (Rosen, 1993). Superoxide dismutase [Cu-Zn] levels were markedly elevated in various neurological diseases, such as bacterial meningitis and encephalitis, and their varied magnitudes may be associated with the underlying diseases (Yoshida et al., 1994). Retinol binding protein 4 (RBP4) is a protein that is encoded in humans by the RBP4 gene (Rocchi et al., 1989). Susceptibility to diet-induced obesity and glucose intolerance in the APP (SWE)/PSEN1 (A246E) mouse model of Alzheimer's disease is associated with increased RBP4 (Mody et al., 2011). Inflammation-dependent cerebral deposition of serum amyloid, a protein, was found in a mouse model of amyloidosis. Inflammation plays an important role in the process of amyloid deposition, and inhibition of inflammatory cascades may attenuate amyloidogenic processes, such as Alzheimer's disease (Guo et al., 2002). Chromogranin A, also called parathyroid secretory protein 1, is a member of the granin family of neuroendocrine secretory proteins, which is encoded by the CHGA gene (Helman et al., 1988). Elevated levels of Chromogranin A in CSF from patients were associated with Astrocytes of Multiple Sclerosis White Matter Lesions (van Luijn et al., 2016).

Nine down-regulated proteins (Rock, Clathrin, Vinculin, Arp2/3, RhoA, Bid, CytC, PKC, ERK) in the CSF of meningitic piglets compared with both non-meningitic and healthy piglets were found and further confirmed by ELISA. Rho/Rock and PKC signaling pathway have been reported to mediated pathogen breaking through the BBB. For example, globotriaosylceramide (Gb3), which interacts with SS2 Fhb to mediate SS2 penetration into the BBB, needs the activation of the Rho/ROCK signaling pathway (Kong et al., 2017). PKC mediates the penetration of *Escherichia coli* K1 and some fungi species in bacterial meningitis (Sukumaran and Prasadarao, 2002; Kim et al., 2012; Salmeri et al., 2012). Vinculin is associated with focal adhesion and adherens junctions (Xu et al., 1998) which are important for maintenance of BBB function. The loss of vinculin impacts a variety of cell functions; it can disrupt the formation of the complex, and prevents cell adhesion and spreading. The absence of vinculin demonstrates a decrease in cell spreading (Goldmann and Ingber, 2002). However, there are no reports about the relationship between clathrin/vinculin and meningitis. Cytoskeleton rearrangement is important for the maintenance of BCB and BBB function. The Arp2/3 complex is a seven-subunit protein complex that plays a major role in the regulation of the actin cytoskeleton and is found in most actin cytoskeleton-containing eukaryotic cells (Veltman and Insall, 2010). Arp2/3 complex-regulated actin rearrangement mediates methamphetamine-induced occludin endocytosis (Park et al., 2013). Previous reports indicate that apoptosis is involved in bacterial meningitis (Xu et al., 2017a,b). In this study, Bid and CytC were down-regulated in the CSF of meningitic piglets compared with both non-meningitic and healthy piglets. Bid, the BH3 interacting-domain death agonist, is a pro-apoptotic member of the Bcl-2 protein family (Wang et al., 1996). Cytochrome c (cytC) is an electron-transfer protein that possesses a wide range of properties and functions in a large number of different redox processes (Moore et al., 1986). Bid and CytC are important regulators of apoptosis (Chipuk et al., 2012). This suggests that apoptosis may play a role in the process of SS2 breakthrough BCB or BBB.

Furthermore, 23 differential proteins (genes name: ATP5A1, ATP5B, ATP5F1, COX5B, COX6B, CYCS, LOC100524613, LOC100525869, LOC733678, NDUFA10, NDUFA12, NDUFA8, NDUFA9, NDUFB10, NDUFB4, NDUFC2, NDUFS1, NDUFS2, NDUFS3, NDUFS7, NDUFV1, SDHA, SDHB) which are involved in brain diseases such as Huntington's disease, Parkinson's disease, and Alzheimer's disease (Figure 5) were selected. This suggests that there may be a common mechanism between these brain diseases and meningitis. But the role of these proteins in process of SS2 meningitis need to be further investigated.

The present work is the first study, to our knowledge, to analyze the differential protein profiling of CSF in SS2 meningoencephalitic pigs compared with both non-meningoencephalitic pigs and healthy pigs. The data provide a theoretical basis for diagnosis, pathogenesis and drug therapy research of SS2 CNS infection. Further studies need to clarify the role of these differential proteins in the process of SS2 meningoencephalitis, though some of these proteins have been reported to be associated with neurological diseases.

## Author contributions

Designed the experiments: LL. Performed the experiments: HL, LJ, YS, RZ, WG, SL, and GQ. Conducted the analysis: HL, LJ, HJ, and JW. Provided the analysis tools and technical support: JG, CS, XF, and WH. Wrote and revised the manuscript: HL and LJ. All authors read and approved the final manuscript.

### Conflict of interest statement

The authors declare that the research was conducted in the absence of any commercial or financial relationships that could be construed as a potential conflict of interest.
